# Improvement of neuropathology and transcriptional deficits in CAG 140 knock-in mice supports a beneficial effect of dietary curcumin in Huntington's disease

**DOI:** 10.1186/1750-1326-7-12

**Published:** 2012-04-04

**Authors:** Miriam A Hickey, Chunni Zhu, Vera Medvedeva, Renata P Lerner, Stefano Patassini, Nicholas R Franich, Panchanan Maiti, Sally A Frautschy, Scott Zeitlin, Michael S Levine, Marie-Françoise Chesselet

**Affiliations:** 1 Departments of Neurology and Neurobiology, David Geffen School of Medicine, UCLA, 710 Westwood Plaza, Los Angeles, CA 90095, USA; 2 Geriatric Research Education Clinical Center, Greater Los Angeles Healthcare System, Veteran's Administration, LA, CA 91343, USA; 3 Departments of Medicine and Neurology, UCLA, Los Angeles, CA 90095, USA; 4 Mental Retardation and Research Center, Semel Neuroscience Institute, Psychiatry and Biobehavioral Science, David Geffen School of Medicine, UCLA, Los Angeles, CA 90095, USA; 5 Department of Neuroscience, University of Virginia, Charlottesville, VA 22908, USA; 6 Current address: Department of Pharmacology, University of Tartu, Tartu 50411, Estonia

**Keywords:** Huntingtin aggregates, Open field, Climbing, Pole task, Rotarod, Grip strength, Striatal mRNA transcripts, Preclinical therapeutic trial

## Abstract

**Backgound:**

No disease modifying treatment currently exists for Huntington's disease (HD), a fatal neurodegenerative disorder characterized by the formation of amyloid-like aggregates of the mutated huntingtin protein. Curcumin is a naturally occurring polyphenolic compound with Congo red-like amyloid binding properties and the ability to cross the blood brain barrier. CAG140 mice, a knock-in (KI) mouse model of HD, display abnormal aggregates of mutant huntingtin and striatal transcriptional deficits, as well as early motor, cognitive and affective abnormalities, many months prior to exhibiting spontaneous gait deficits, decreased striatal volume, and neuronal loss. We have examined the ability of life-long dietary curcumin to improve the early pathological phenotype of CAG140 mice.

**Results:**

KI mice fed a curcumin-containing diet since conception showed decreased huntingtin aggregates and increased striatal DARPP-32 and D1 receptor mRNAs, as well as an amelioration of rearing deficits. However, similar to other antioxidants, curcumin impaired rotarod behavior in both WT and KI mice and climbing in WT mice. These behavioral effects were also noted in WT C57Bl/6 J mice exposed to the same curcumin regime as adults. However, neither locomotor function, behavioral despair, muscle strength or food utilization were affected by curcumin in this latter study. The clinical significance of curcumin's impairment of motor performance in mice remains unclear because curcumin has an excellent blood chemistry and adverse event safety profile, even in the elderly and in patients with Alzheimer's disease.

**Conclusion:**

Together with this clinical experience, the improvement in several transgene-dependent parameters by curcumin in our study supports a net beneficial effect of dietary curcumin in HD.

## Background

Huntington's disease (HD) is an autosomal dominant neurodegenerative disorder caused by an elongated, unstable, polyglutamine repeat near the N terminus of the *huntingtin *gene [1]. Recent studies have shown that many symptoms including behavioral, cognitive and motor changes are present in gene carriers decades prior to the clinical onset of the disease [2,3]. Further, pathological changes including striatal atrophy, cortical thinning and white matter loss, aggregates of mutant huntingtin, receptor loss and microgliosis are present many years prior to predicted age of disease onset [4-10]. Therefore neuroprotective treatments may need to be started in gene carriers long before the onset of manifest disease [7]. This requires the use of drugs with an excellent safety profile over long periods of administration. Moreover, it is possible that this early drug treatment could prevent later downstream toxicity due to the huntingtin protein.

CAG140 knock-in (KI) mice are a slowly progressing mouse model of HD that exhibit pathological, molecular and behavioral deficits as early as 2 years before developing spontaneous motor deficits which is itself reminiscent of the clinically manifest phase of HD [11,12]. These mice express a chimeric human/mouse Hdh protein, including human mutant exon1 with approximately 140 CAG repeats. When they begin to show obvious anomalies in homecage behavior around 2 years of age, these mice show 38% loss in striatal volume and 40% striatal neuronal loss, remarkably similar to the 1/3 to 1/2 loss in HD patients at pheno-conversion [4]. In CAG140 mice, this is preceded by stride deficits, neurochemical anomalies, cortical gliosis and cortical and striatal electrophysiological changes at 12 months of age [11-14]. However, deficits in open field, climbing, sensorimotor activity, wheel running, motor learning, and anxiety, as well as pathological accumulation and aggregation of huntingtin in the nucleus and cytoplasm are typically present prior to 6 months of age, with some anomalies occurring as early as 1 month of age [11,12]. In addition, reduced actin polymerization, abnormal long term potentiation, and deficits in long-term novel object recognition memory are present by 4 months in these mice [15]. Thus, the CAG 140 KI mice provide an excellent opportunity to study and treat the earliest changes induced by the mutant protein.

Curcumin, a major bioactive component of turmeric, has multiple pharmacological properties and has shown beneficial effects in *in vivo *models of aging, ischemia and trauma [16-19]. In addition to its anti-inflammatory and antioxidant activities, curcumin is a Congo red-like agent with anti-aggregate properties, and it can cross into brain parenchyma as shown by measurement in brain tissue following administration in the diet [20-22]. Its anti-amyloid properties and oral availability suggest that it may be a promising compound for the treatment of several neurodegenerative diseases. Indeed, in mouse models of Alzheimer's disease, curcumin and related curcuminoids reduced plaque burden and microglia, improved cognitive function, and protected against Aβ toxicity *in vitro *and *in vivo *[20,23-26]. Further, its low toxicity demonstrated by millennia of use as a food additive makes curcumin an attractive possibility for the early and chronic treatment of neurodegenerative diseases.

Here, we tested the hypothesis that the Congo-red-like properties of curcumin could be exploited to reduce the early neuropathology in CAG140 KI mice, without the toxicity and need for intracerebral delivery associated with Congo red administration [27]. To determine whether any effect on pathology was ultimately beneficial for cellular function, we measured a panel of striatal transcripts known to be altered by transcriptional dysregulation resulting from mutated huntingtin expression [28]. Although our *in vitro *studies here show that dosing is important, as others have shown [20,29], safety and toxicity studies *in vivo *have repeatedly shown that curcumin has a very favorable safety profile [30,31]. Curcumin is an anti-oxidant and induces antioxidant response elements [29,32]. Although oxidative damage can mediate pathogenesis in neurodegenerative diseases, redox balance is important for several aspects of physiology including learning and memory, and normal cellular function [33]. Therefore, we conducted a study of motor behavior in WT and KI mice treated from conception as well as a study in WT mice treated as adults.

## Results

WT, KI and HET mice were administered curcumin via chow at a continuous dose of 555 ppm. HET mice were not behaviorally tested, but were perfused with PBS at the end of treatment (identical, parallel treatment with WT and KI mice) for curcumin measurement in blood-free brain tissue. As expected from previous studies with the same regimen in mouse models of Alzheimer's disease, curcumin levels in brain tissue were in the nanomolar range (Table 1). No curcumin was detected in control-treated mice (Table 1). Curcumin levels were slightly elevated in fresh frozen tissue, reflecting blood content (Table 1). Thus, this regimen provided therapeutic levels of curcumin in brain tissue based on evidence that a very similar curcumin regimen was beneficial in mouse models of Alzheimer's disease [20,25,26]. A dose of 555 ppm (555 mg/kg food; 92 mg/kg mouse body weight; calculations based on [20]) correlates to a dose of approximately 7.1 mg/kg human body weight or 625 mg curcumin per day based on the recommended correlation to body surface area (http://www.accessdata.fda.gov/scripts/cder/onctools/animalquery.cfm and [34]; using a weight of 87 kg [US male average] and height of 177.6 cm [US male average] and mouse weight of 30 g). Although no study to date has measured curcumin levels in the brain of patients treated with curcumin, the dose used in this study is well below doses (1 or 4 g/d) used for 6 months in elderly patients without adverse effects on blood chemistries or other serious adverse side effects [30,35].

**Table 1:** Curcumin levels in whole brain from curcumin-treated mice

Treatment	Fresh frozen (nM)	PBS-perfused (nM)
Curcumin	174 ± 17	128 ± 8*

No curcumin was detected in control-treated tissue (n = 2 WT fresh frozen, n = 4 HET PBS-perfused). Data are mean ± sem. Group sizes are 3 (WT, fresh frozen) and 5 (HET, PBS-perfused). *p < 0.05 compared to fresh frozen.

Curcumin is a non-flavonoid polyphenolic and as both flavonoids and non-flavonoids can cross the placenta [36,37] it was administered from conception. In agreement with previous studies [38,39] we found no difference in either the number of pups born or number of pups weaned between control-fed and curcumin-fed dams (Table 2, effect of food, F(1,18) = 0.39, ns, interaction between food and age of pups (birth to weaning), F(1,18) = 1.59, ns). In addition, there was no difference between groups in the percentage of pups brought through to weaning per dam (Table 2, Student's *t *test, ns). Thus, as expected, we found no deleterious effects of dietary curcumin on fecundity.

**Table 2:** Reproductive success of heterozygous CAG140 dams treated with curcumin

Treatment	Pups/dam born	Pups/dam weaned	Percent of pups brought through to weaning
Control	6.3 ± 0.45	5.8 ± 0.7	94 ± 12

Curcumin	6.6 ± 0.43	4.7 ± 0.9	81.1 ± 16

Data are mean ± sem.

**Figure 1:**
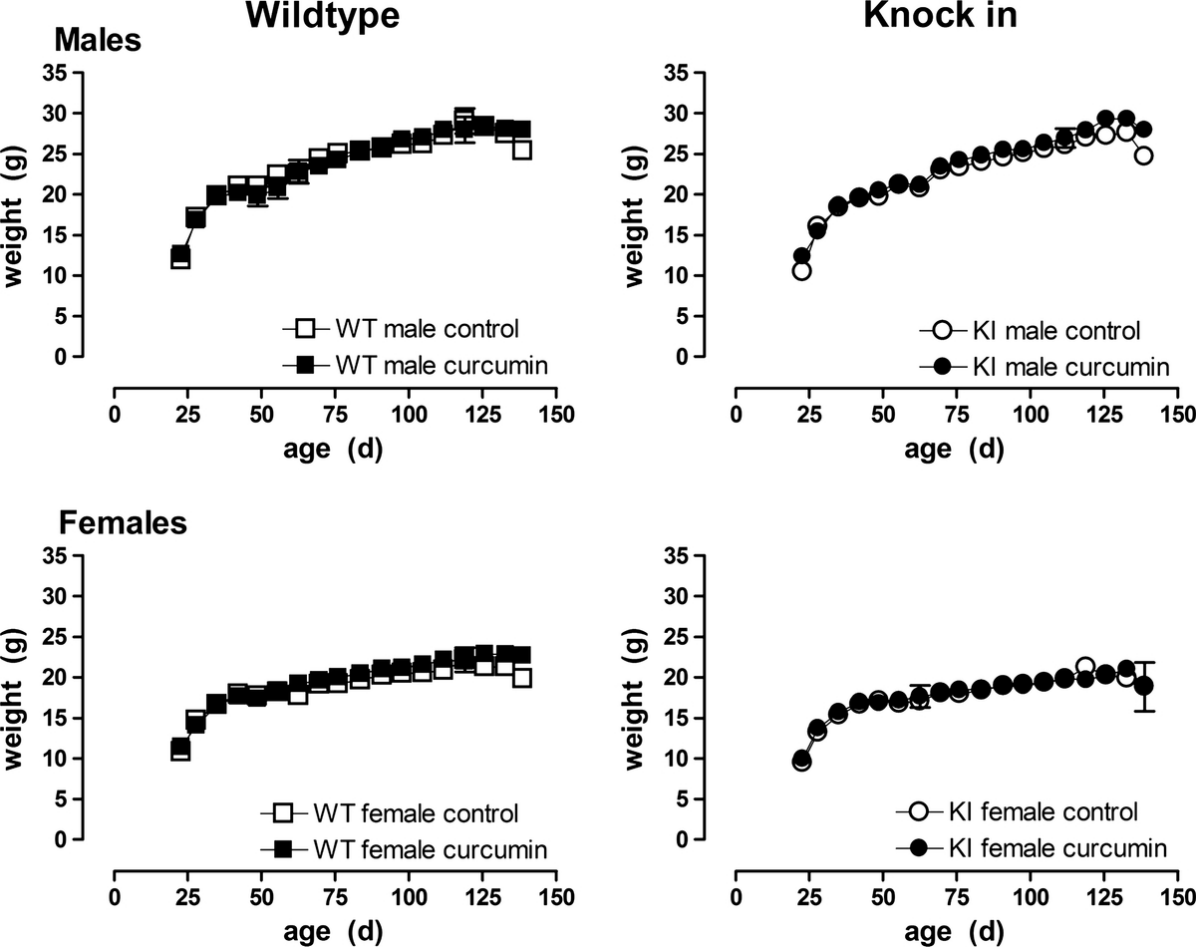
**Body weight profiles of WT and KI mice fed curcumin or control chow**. No difference was detected between curcumin-fed and control-fed male mice, and while an overall effect of genotype was detected in female mice, no effect of food, or interaction of food and genotype, or food and genotype and age were found in either gender, indicating, as expected, no deleterious effect of curcumin. Data shown are mean ± sem. WT male n = 13-15; WT female n = 15-19; KI male n = 7-10; KI female n = 7-9. Data analyzed using GLM ANOVA followed by post hoc Bonferroni t-tests.

**Figure 2:**
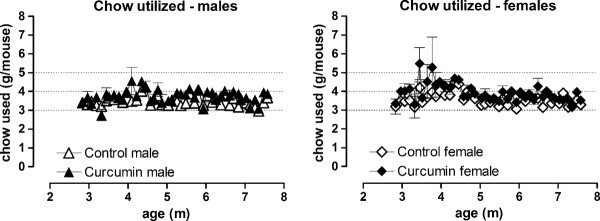
**Chow utilized by control-fed and curcumin-fed adult C57Bl/6 J mice**. No difference was noted between the groups. Data are mean ± sem. N = 9-10 per group.

We have previously shown that CAG140 KI mice show normal body weight gain up to 7 m of age, with reduced body weight noted from 1 y of age [40]. Because our goal was to examine the effects of curcumin on the earliest manifestations of mutant huntingtin, mice were euthanized at 4.5 m. At this age CAG140 KI mice typically show robust neuropathological changes and behavioral deficits, however overt spontaneous changes do not occur until 2 years of age [11,12]. Weight was monitored in WT, KI and Het mice. In keeping with known toxicological data on curcumin, male body weight was normal and was unaffected by curcumin (males, genotype × food × age F(34,812) = 0.63, ns). There was an overall effect of genotype in female mice although post hoc testing showed no between-groups differences at any age (effect of genotype F(2,62) = 4.71, p < 0.02), and again there was no effect of food (F(1,62) = 2.2, ns) or interaction between food, genotype and age (genotype × food × age F(34,913) = 0.7, ns). Therefore, curcumin did not appear to have any deleterious effects on general health (Figure 1; only WT and KI data are shown). We did not measure food utilization in this group of mice, however in a subsequent trial of similar length, conducted in normal adult C57Bl/6 J mice, food utilization was similar between groups, indicating that palatability did not differ between treated and untreated chow (3-4 g eaten per mouse per day; Figure 2, food × age interaction, males: F(42, 168) = 1.43, ns; females: F(42,168) = 0.69, ns).

### *In vitro *and *in vivo *neuropathological analysis

**Figure 3:**
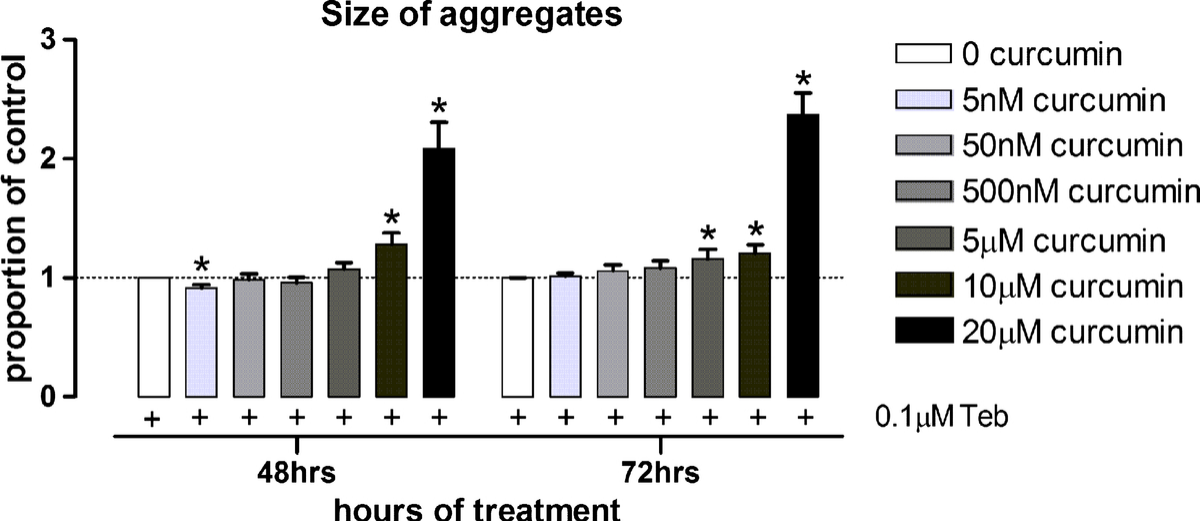
**At nanomolar concentrations, curcumin slightly reduces aggregate size and at micromolar concentrations, curcumin increases aggregate size in PC12 cells inducibly expressing exon 1 of mutant htt **[42]. Cells were induced with 0.1 μM tebufenozide and treated with curcumin (5 nM, 50 nM, 500 nM, 5 μM, 10 μM or 20 μM) or vehicle (DMSO) at the same time. Uninduced cells (treated with EtOH (vehicle) were used as a control for expression of the mutant protein) are not shown. At 48 h, cells treated with 5 nM curcumin show an 8% reduction in size of aggregates compared to vehicle-treated induced cells (no curcumin). At 48 h and at 72 h, 10 or 20 μM increase aggregate size markedly, possibly reflecting curcumin's effect on the UPS at micromolar concentrations [43]. Data shown are of the mean ± sem of n = 4 independent experiments.

A primary goal of the experiment was to determine whether curcumin, which has anti-amlyoid properties, could affect protein aggregates of huntingtin in the striatum of CAG140 KI mice. High concentrations of curcumin increases aggregate size *in vitro *[41] and indeed, using PC12 cells that inducibly express EGFP-tagged exon 1 of mutant htt [42], we found that 10 or 20 μM curcumin, applied at the same time as protein induction, increased aggregate size markedly at 48 or 72 h post mutant-protein induction (Figure 3; 48 h, Kruskal-Wallis ANOVA p < 0.01, *p < 0.05 compared to control treated cells; 72 h, Kruskal-Wallis ANOVA p < 0.01, *p < 0.05 compared to control treated cells). In contrast to 20 μM curcumin, 5 nM curcumin caused a slight reduction in aggregate size at 48 h (Figure 3; 48 h, Kruskal-Wallis ANOVA as above, *p < 0.05 compared to control treated cells) but the effect was very small (-8% reduction in size of all aggregates) and was only observed with the lowest concentration tested, which is well below the concentration of curcumin measured in brain in our study (Table 1).

Despite the lack of clear effects of low concentrations of curcumin *in vitro *on aggregate sizes, we observed a clear decrease in aggregate numbers in the striatum of KI mice after administration of curcumin *in vivo*. Only tissue from KI mice was examined because huntingtin-stained nuclei and aggregates are not detected in the brain of WT mice [12]. An histological approach to analyzing aggregates was chosen to allow for the separate analysis of several types of aggregates; in addition, we performed a regional analysis by examining two levels of striatum, and the level of striatum was included as a factor in the ANOVA analyses because it had a significant effect on the distribution of neuropil aggregates (F(1,20) = 9.74, p < 0.01), diffusely stained nuclei (F(1,20) = 25.37, p < 0.0001) and microaggregates (F(1,20) = 29.76, p < 0.0001), independent of treatment. Importantly, power calculations show that a group size of 2-3 mice are sufficient to detect a 30% change in the number of stained nuclei, or number of nuclei containing microaggregates (α = 0.05, 80% power), thus, our group sizes were well powered to detect treatment effects.

**Figure 4:**
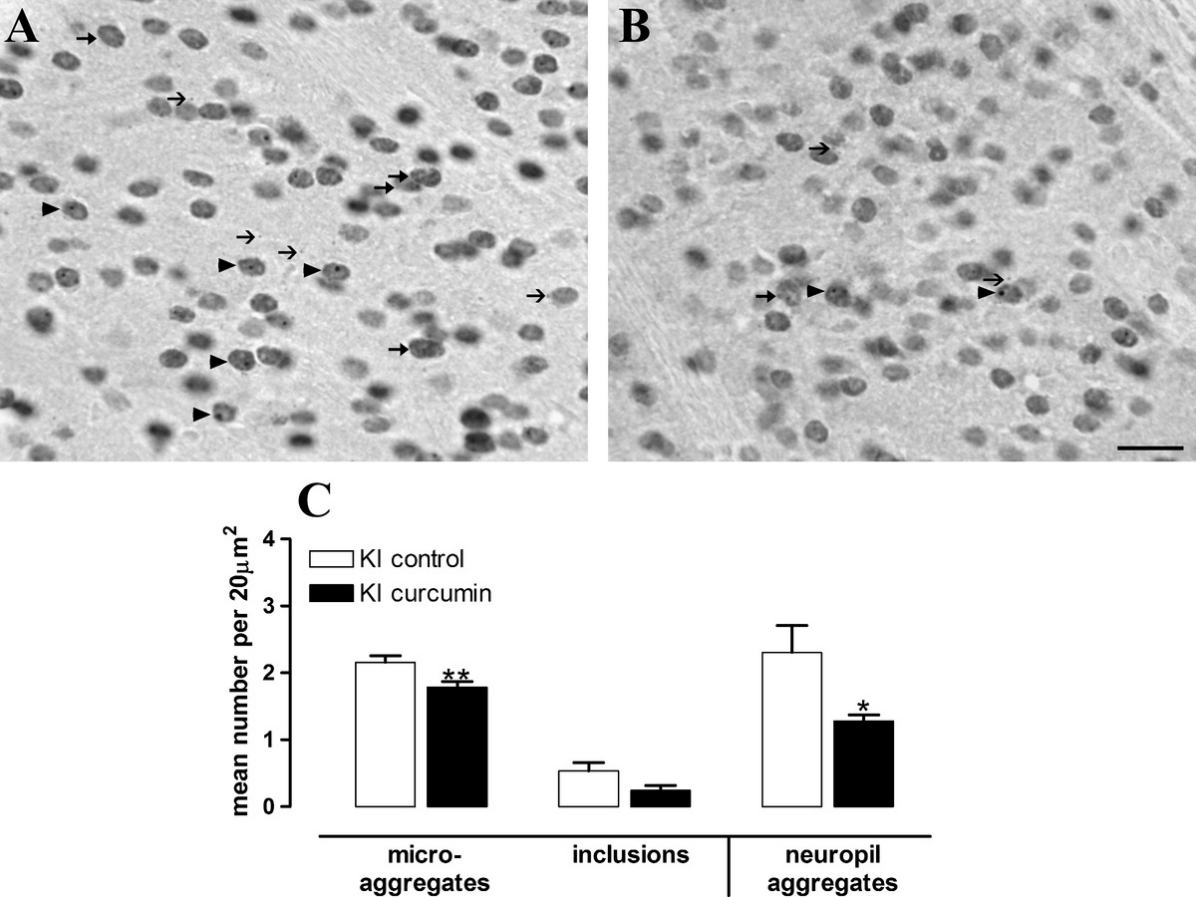
**Curcumin reduces aggregates in KI mice**. Mice were fed curcumin from conception and were analyzed for huntingtin aggregates, at 4.5 m of age. The mean number of stained nuclei (data not shown), stained nuclei containing microaggregates or inclusions, and neuropil aggregates per 20 μm^2 ^were counted over the entire striatum of each of two sections per mouse (1.32 mm and 2.28 mm lateral of the midline [47], data from medial section shown). A) control-treated KI mouse, B) curcumin-treated KI mouse. Arrowheads indicate stained nuclei containing inclusions. Small line arrows indicate neuropil aggregates and arrows indicate stained nuclei with microaggregates. C) Quantification of neuropathological analysis. Curcumin reduces several forms of aggregated huntingtin in 4.5 m old KI mice, data from medial section shown. Although the reduction in number of inclusions is not significant, a strong trend towards reduction was observed (effect of treatment F(1,20) = 4.3, p = 0.052). Data are shown as mean ± sem and were analyzed using ANOVA followed by Fishers LSD post hoc tests. N = 6 per group. * p < 0.05, **p < 0.01, compared to control-treated KI. Arrows are as for A) and B). Scale bar = 20 μm for A) and B).

At 4.5 m of age, curcumin-fed KI mice had less diffusely stained nuclei, the earliest huntingtin-related pathology observed in mutant mice, than control-fed mice (effect of treatment F(1,20) = 5.02, p < 0.05; Figure 4A, B, C). Curcumin-treated KI mice also showed fewer microaggregates, defined as numerous, small, nuclear puncta, similar to those previously observed in other models of HD [44-46] (effect of treatment F(1,20) = 12.73, p < 0.01; Figure 4A, B, C). Nuclear inclusions were reduced by curcumin but this effect did not reach significance (effect of treatment F(1,20) = 4.3, p = 0.052). By far the largest reduction observed in curcumin-treated mice (-17% in lateral sections, -45% in medial sections) was on neuropil (cytoplasmic) aggregates in the striatum (effect of treatment, F(1,20) = 5.22, p < 0.05, Figure 4A, B, C). These data indicate that curcumin improved a key pathological consequence of the HD-causing mutation *in vivo*.

### Transcriptional changes

**Figure 5:**
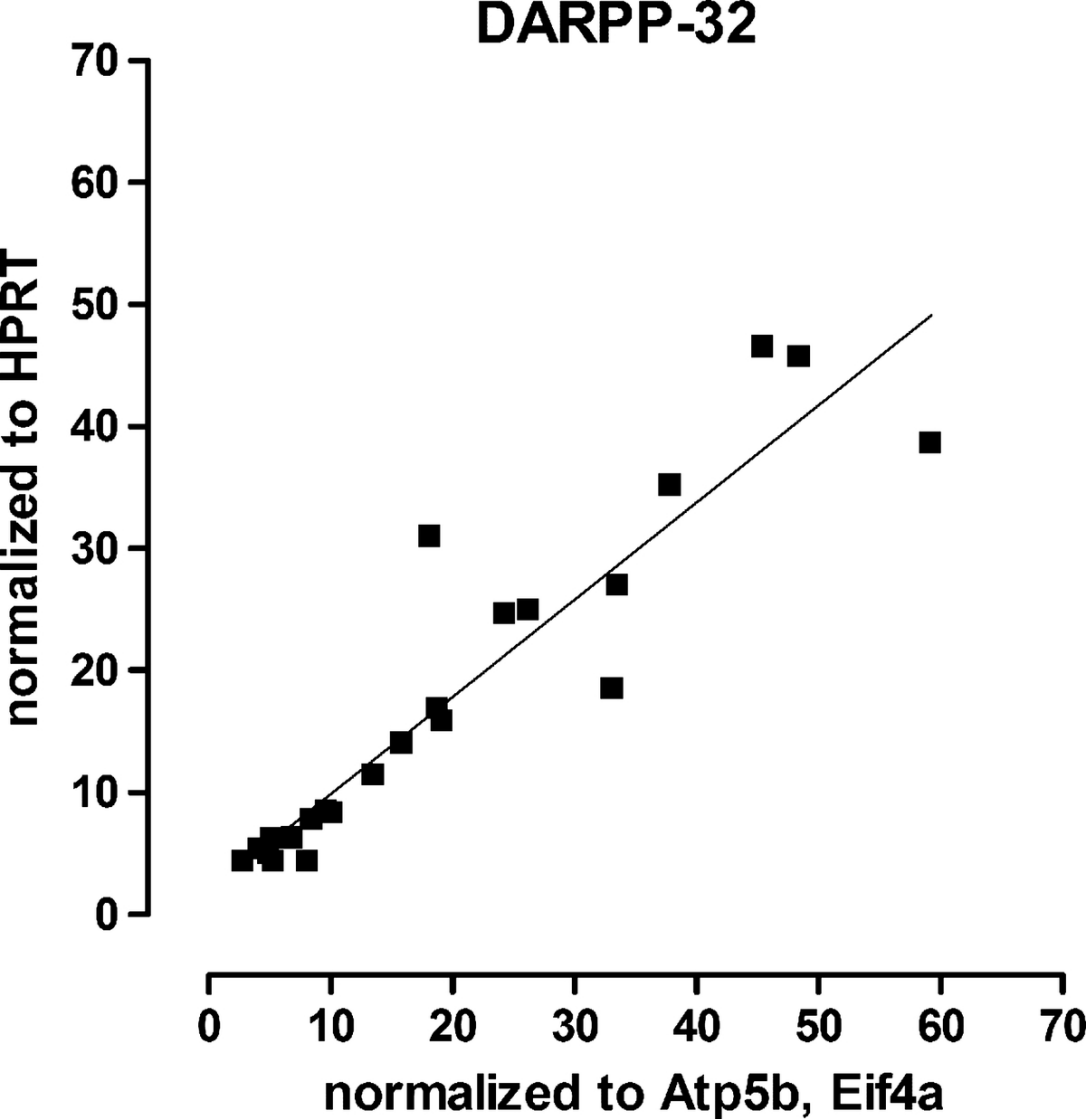
**Correlation and linear regression analysis of levels of striatal DARPP-32 mRNA**. Using a separate group of mice to those used in our curcumin preclinical trial (n = 23 in total), we correlated levels of DARPP-32 mRNA normalized using HPRT or Atp5b and Eif4a [53]. We found excellent correlation between the results obtained with HPRT and with Atp5b and Eif4a (r^2 ^= 0.862 and slope of 1.08, p < 0.0001).

Having established a profound effect on htt aggregation, we went on to assess whether transcription was also improved. Together with the formation of protein aggregates, transcriptional dysregulation is a major effect of mutant huntingtin [48]. We have previously detected profound changes in striatal transcripts for enkephalin but not substance P at 4 months of age in a similar line of KI mice with 94 CAG repeats (CAG94; [28]). We have also detected a significant decrease of D1, D2 dopamine and CB1 receptors, enkephalin and DARPP-32, but not substance P mRNA at 4 months of age in CAG140 KI mice [49]. Power calculations showed that group sizes of 2-7 (depending on transcript) are required to detect a 50% change in the number transcripts in the KI mice (α = 0.05, 80% power), thus our group sizes were well powered to detect treatment effects (n = 7 for each KI group). The housekeeping gene used was HPRT, as used previously [50-52]. In a separate group of mice (n = 23), we found that HPRT showed excellent correlation to results obtained using other housekeeping genes, with r^2 ^= 0.862 and slope of 1.08 (Figure 5, p < 0.0001 [53]).

We confirmed striatal transcriptional dysregulation in the control-fed KI mice compared to WTs (Table 3, effect of genotype: Enkephalin F(1,26) = 78.1, p < 0.0001; D1 F(1,26) = 27.5, p < 0.0001; D2 F(1,26) = 29.6, p < 0.0001; DARPP-32 F(1,26) = 51.7, p < 0.0001; CB1 F(1,26) = 21.8, p < 0.0001; and post hoc tests using Bonferroni's multiple t-tests, corrected for 4 comparisons).

**Table 3:** Effect of curcumin treatment on striatal transcripts

Transcript	Control-treated			Curcumin-treated			Treatment effect in KIs
	WT	KI	Percent change from WT	WT	KI	Percent change from WT	Percent rescue from control-treated KI levels
D1 receptor	1.13 ± 0.13	0.64 ± 0.04*	-43.4	1.1 ± 0.04	0.8 ± 0.03^‡^	-27.4	+25
D2 receptor	1.31 ± 0.12	0.73 ± 0.04**	-44.1	1.07 ± 0.05	0.84 ± 0.04*	-22.1	+14
CB1 receptor	0.93 ± 0.13	0.48 ± 0.07*	-48.5	1.03 ± 0.09	0.61 ± 0.05	-40.9	+26.7
DARPP-32	1.28 ± 0.1	0.61 ± 0.03**	-52.1	1.09 ± 0.06	0.79 ± 0.04**^‡^	-27.4	+29.5
Enkephalin	1.06 ± 0.06	0.55 ± 0.04**	-47.7	0.98 ± 0.06	0.56 ± 0.04**	-43.0	+1.5
Substance P	0.95 ± 0.06	0.99 ± 0.07	+5.1	0.91 ± 0.09	0.95 ± 0.09	+4.7	-4.2

WT n = 8 per group, KI n = 7 per group. * p < 0.05, ** p < 0.01 compared to control-treated WT (comparisons to WT curcumin not shown, for clarity). ^‡ ^p < 0.05 compared to control-treated KI. Raw data were compared using completely randomized ANOVA followed by Bonferroni's corrected for 4 comparisons. Groups were composed of balanced mixed-gender groups. Data were processed as described previously [54].

Curcumin treatment attenuated several deficits in KI mice, with D1 and CB1 mRNA no longer being different to control-fed WT mice, and the levels of DARPP-32 and D1 receptor mRNA being significantly increased compared to control-fed KIs (Table 3). No transcripts were significantly affected in WT mice (Table 3).

### Behavioral analysis

In addition to curcumin's effect on pathological and molecular changes induced by mutant huntingtin we also examined the effects of curcumin on the early behavioral deficits in KI mice. Analyses were carried out at 1 month (open field), 1.5 month (climbing) and 4 months of age (pole task and rotarod). Several behavioral deficits are typically present in these tests at these ages in the KI mice, indicative of the extensive neural dysfunction present at these early ages [11,12,49].

For mice treated with curcumin or control diets from conception, there was no effect of gender on any of the tasks examined (effect of sex: for open field first 5 mins F(1,85) = 3.6, ns; climbing: F(1,80) = 3.1, ns; pole task: F(1,77) = 0.003, ns; rotarod 10 rpm: F(1,81) = 2.8, ns, 20 rpm, F(1,81) = 0.21, 30 rpm F(1,81) = 0.92, ns; no interaction between genotype, treatment and sex: for open field: first 5 mins, F(1,85) = 2.1, ns); climbing: (F(1,80) = 1.4, ns); pole task: (F(1,77) = 2.6, ns) or rotarod (10 rpm: F(1,81) = 0.03, ns; 20 rpm: F(1,81) = 0.38, ns; 30 rpm, F(1,81) = 0.6, ns). Therefore, data from males and females were grouped for analysis.

**Figure 6:**
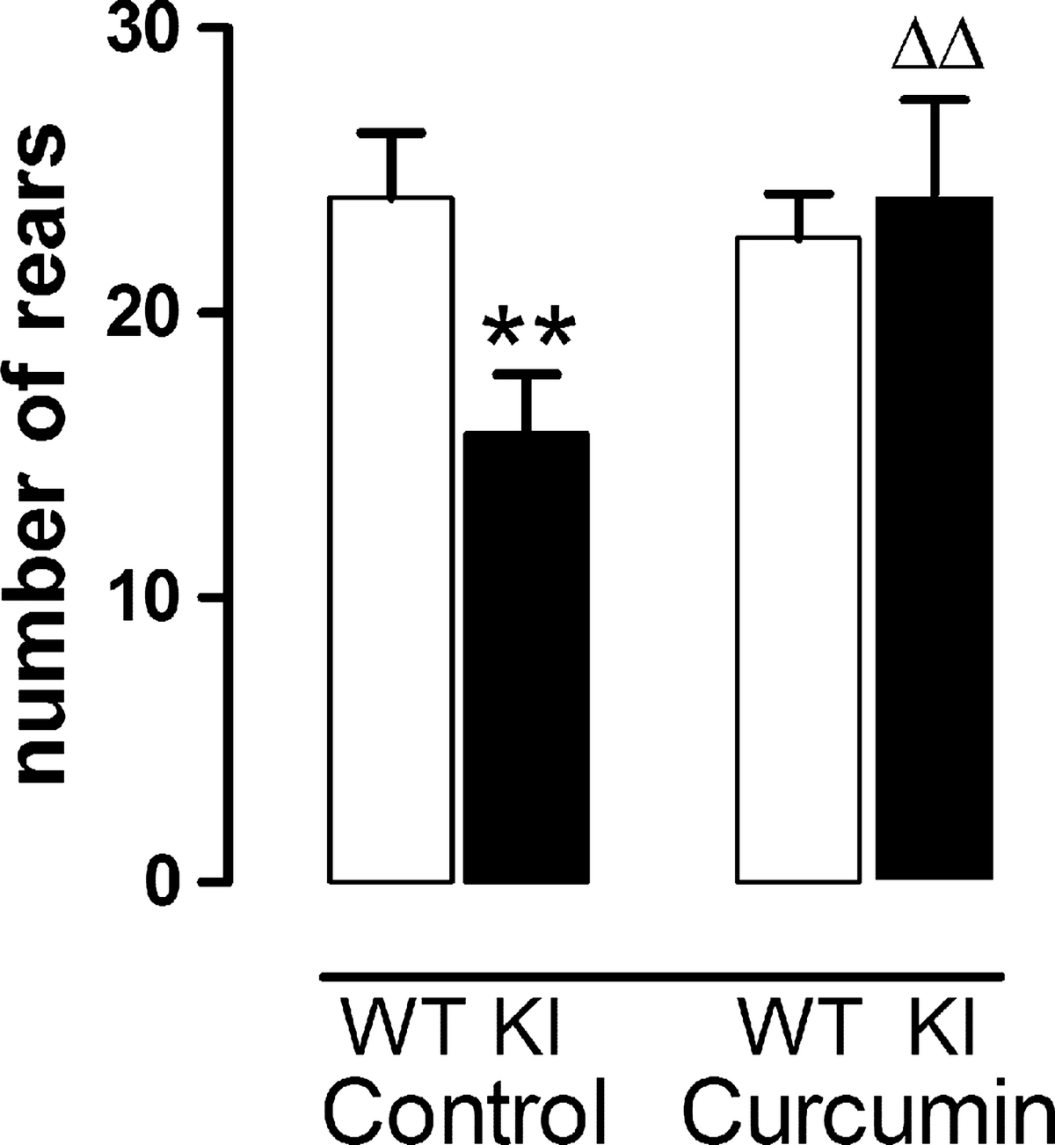
**Curcumin rescues rearing deficit in KI mice**. Control KI mice rear less than their littermate WT controls and treatment with curcumin abolished this deficit. Data are mean ± sem of first 5 min in open field. ** p < 0.01 compared to control-fed WTs, ΔΔ p < 0.01 compared to control-fed KI group. Groups were composed of balanced mixed gender groups since there was no significant effect of gender (see text). Data were analyzed using ANOVA followed by Fishers LSD post hoc tests. WT n = 29-32, KI n = 16 per group.

Curcumin treatment rescued the reduced rearing in KI mice observed in the first five min in the open field (genotype × treatment F(1,89) = 4.36, p < 0.05, Figure 6). As previously shown [11], climbing was decreased at 1.5 months in KI mice (Table 4, effect of genotype F(1,84) = 4.6, p < 0.05; no overall interaction between genotype and treatment F(1,84) = 3.1, ns). Post hoc analysis showed that compared to control diet curcumin abrogated the difference between KI and WT curcumin-fed mice suggesting a beneficial effect of treatment. However, curcumin impaired climbing in WT mice (see Table 4). This suggests that the effect of curcumin on HD pathogenesis may be stronger than, and opposite to, the "off-target" effects in WT mice.

**Table 4:** Effect of curcumin treatment on climbing behavior and pole task performance in CAG140 KI and WT mice

	Number of climbs (1.5 m)		Time taken to turn (4 m, (s))	
	**WT**	**KI**	**WT**	**KI**

Control-treated	1.21 ± 0.33	0.14 ± 0.1**	3.01 ± 0.35	5.22 ± 0.84**

Curcumin-treated	0.48 ± 0.17*	0.38 ± 0.18*	3.03 ± 0.32	4.06 ± 0.69

WT n = 26-30, KI n = 14-16, * p < 0.05, ** p < 0.01 compared to control-treated WT (comparisons to WT curcumin not shown, for clarity). Groups were composed of balanced mixed gender groups as there was no significant effect of gender (see text).

**Figure 7:**
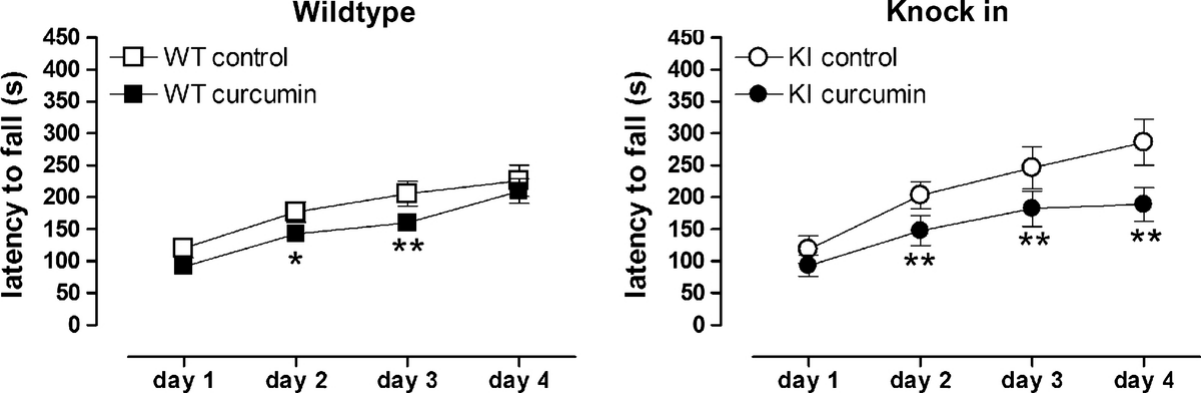
**Although at this age no rotorod deficits were detected in the KI mice relative to WT, curcumin lowered performance scores**. At the end of the trial, when tested on the rotarod, both WT (left) and KI (right) mice treated with curcumin showed impaired performance. Data shown are mean ± sem. Groups were composed of balanced mixed gender groups since there was no significant effect of gender (see text). Data were analyzed using ANOVA followed by Fishers LSD post hoc tests. WT n = 28-30, KI n = 15-16 per group. *p < 0.05, ** p < 0.01 compared to genotype-matched controls on the same day.

As previously shown [11], 4 month old control-fed KIs were impaired on the pole task (Table 4, effect of genotype F(1,81) = 10.1, p < 0.01). Curcumin-treated KIs were no longer significantly different from WT controls, indicating a small beneficial effect (Table 4). In agreement with other studies of rotarod performance of knock-in mice [55], we have previously shown that impairments on the rotarod in CAG140 KI mice are very subtle, with no impairments during accelerating protocols and small impairments during fixed speed protocols [11]. In the present study, control-treated KIs showed no defects in rotorod peformance, and actually performed slightly better than WTs on training days 3 and 4. However, curcumin impaired performance in both WTs and KIs throughout training. The curcumin-treated mice appeared to learn the task during training, since all mice improved over successive days, having started out with similar performances (see Figure 7, day 1). However, over time, both control-fed WTs and control-fed KIs showed improved performance above that of their genotype matched curcumin-fed counterparts (Figure 7, training: days 1 through 4, accelerating paradigm, smooth axle, effect of treatment F(1,86) = 9.2, p < 0.01, no interaction of genotype and treatment F(1,86) = 1, ns). The effect of treatment was also seen after the training phase, on day 5 with the smooth axle fixed speed paradigm (effect of treatment F(1,85) = 12.6, p < 0.001; for example at 20 rpm: WT control 139 ± 39 s, WT curcumin 37 ± 7, KI control, 182 ± 64, KI curcumin 34 ± 9, WT control versus WT curcumin p < 0.01, KI control versus KI curcumin p < 0.01, Fishers LSD post hoc, no interaction of genotype and treatment F(1,85) = 0.5, ns), and on day 6 with the grooved axle (effect of treatment F(1,85) = 16.3, p < 0.001; for example 30 rpm WT control 274 ± 47 s, WT curcumin 81 ± 26, KI control, 248 ± 68, KI curcumin 91 ± 40, WT control versus WT curcumin p < 0.01, KI control versus KI curcumin p < 0.01, Fishers LSD post hoc; no interaction of genotype and treatment F(1,85) = 0.6, ns; 1 WT control mouse died before days 5 and 6).

To determine whether the deleterious effects of curcumin in WT mice on climbing and rotarod performance were related to exposure to curcumin during early development, we conducted a separate trial of curcumin in normal C57Bl/6 J WT mice. Treatment began at 2.7 months and continued until 8 months of age, to match the total length (gestation and postnatal) of treatment used in the CAG140 trial (approximately 23 weeks).

**Figure 8:**
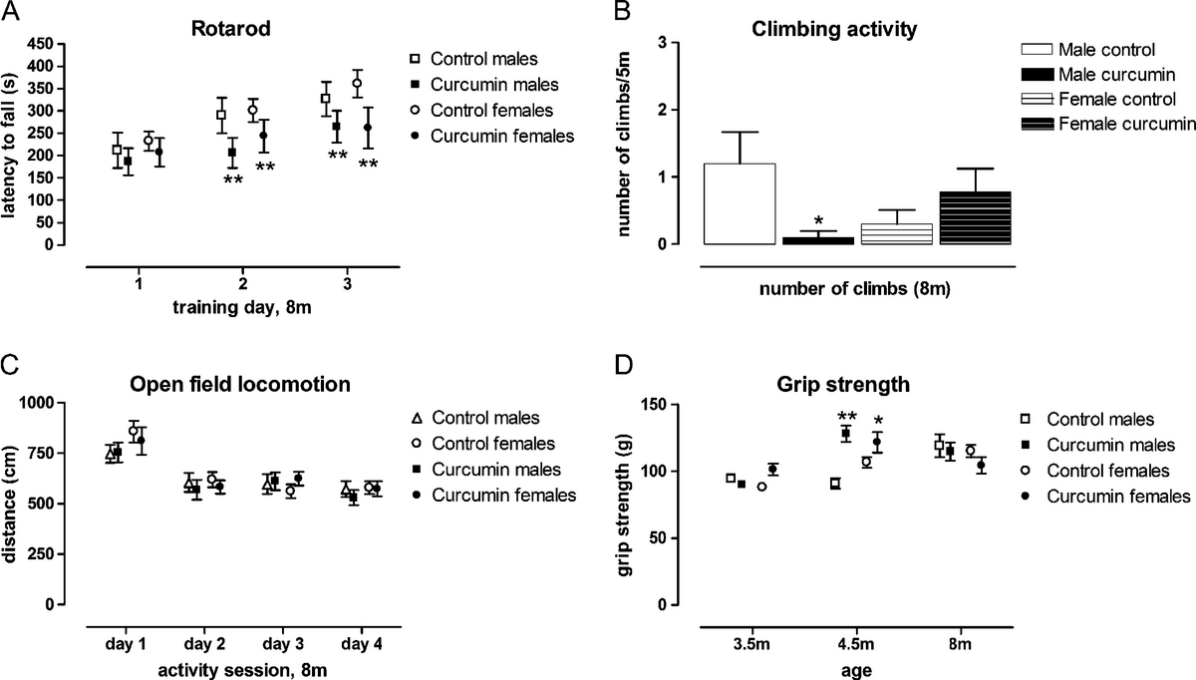
**Curcumin impairs rotarod performance in normal C57Bl/6 J mice, but does not affect forelimb muscle strength, or locomoter activity**. Adult mice were fed from 2.7 m until 8 m of age with curcumin, to approximate the duration that CAG140 mice were fed (gestation + neonatal + adult). Curcumin impaired performance in the rotorod in a gender-independent manner (A). At 8 m age curcumin treatment of males by not females was associated with climbing deficits. Overall, females climbed less than males possibly obscuring treatment effects (B). Curcumin improved grip strength at 4.5 m age, but did not affect grip at other ages tested (C). D) Activity in the open field was normal. Data are shown as mean ± sem, n = 10 per group (n = 9 in curcumin-fed females). Data were analyzed using ANOVA followed by Fishers LSD post hoc tests. *p < 0.05, **p < 0.01 compared to control-fed gender-matched mice.

As in the life-long curcumin trial, both male and female curcumin-fed WT mice were impaired in rotarod performance by endpoint (Figure 8A, 8 months, treatment × training session F(2,70) = 4.1, p < 0.03). We did not observe curcumin-induced impairments in climbing until 8 months of age. This deficit was only in males, however, climbing activity was reduced in all females at this age, possibly obscuring any treatment effects (Figure 8B, gender × treatment interaction at 8 months, F(1,35) = 6.2, p < 0.02). The reduction in rotarod performance was not due to a general effect of curcumin on movement, since there was no effect of curcumin on distance moved in the open field (Figure 8C, effect of day F(3,105) = 36.7, p < 0.0001, treatment × day interaction F(3,105) = 0.93, ns). Grip strength was actually increased by curcumin treatment and then normalized by the endpoint (Figure 8D). As expected, gender impacted muscle strength, thus, the analysis was conducted separately in males and females (Figure 8D, treatment × gender × time, F(2,70) = 4.3, p < 0.02). These data demonstrate that muscle strength was not impaired in curcumin-treated mice, and as such does not explain the impaired rotarod performance. Latency to immobility was unchanged between treatment groups in the forced swim task indicating that behavioral despair, also, was not the cause of the impaired rotarod performance (data were analyzed separately in males and females since gender impacted levels of immobility, latency to immobility: treatment × gender F(1,35) = 0.26, ns; data not shown).

Taken together, these data in adult WT mice demonstrated that fine motor coordination, muscle strength, depressive behavior and activity levels were not the cause of impaired rotarod or climbing performance induced by curcumin treatment. Nevertheless these deleterious effects are not unexpected, since CoQ10 is an anti-oxidant and component of the electron transport chain, also impairs rotarod performance in WT mice [56,57].

## Discussion

Curcumin, a component of turmeric, has beneficial effects in animal models of several types of neurodegenerative diseases [16-20,23-26]. Indeed, clinical trials with curcumin are underway for mild cognitive impairment and Alzheimer's disease http://www.clinicaltrials.gov. Curcumin has many reported properties including dose dependent effects on protein aggregation and transcription, as well as anti-inflammatory and anti-oxidant effects [20,31]. Importantly, curcumin is safe in clinical tolerability trials even in elderly AD patients [30,35]. It labels amyloid plaques in the brain of mouse models of AD *in vivo *and *ex vivo *following i.p. or oral administration [24,26] and fibrillar intracellular tau in human AD pathological samples [58]. Curcumin is capable of inhibiting aggregation and disaggregating Aβ *in vitro *and this depends on the fibril-related conformation rather than sequence [26]. Thus, the many properties of curcumin indicate its potential for HD.

We treated WT and CAG140 KI mice, a genetically accurate model of HD, with 555 ppm curcumin in their chow from conception in order to expose the mice to the agent for as long as possible. We chose this dose because higher doses may actually be less beneficial due to the demonstrated toxic effects of micromolar levels of curcumin in an *in vitro *model of HD [41] and lesser efficacy in reducing amyloid burden in the Tg2576 mouse [25]. At micromolar concentrations, curcumin inhibits the proteasome, which may exacerbate the disease, and increased aggregate sizes *in vitro *in our study. Therefore, we chose a low concentration that has been previously shown to produce beneficial effects in mouse models of AD [20,25,26]. This dose is much lower than doses previously shown to be safe in a 6 months treatment in elderly patients [35].

Free (unglucuronidated) curcumin can cross the blood-brain barrier following oral administration [20,21,26,59] and is detected in blood-free mouse brain parenchyma following oral and systemic administration at one hour after last dose in an acute dosing (1 gavage per day, for 2 days) regimen [20]. After initial loading, curcumin is very stable in lipid environments, and after chronic dosing produces constant levels in the blood [20]. Plasma levels in Tg2576 after several months dosing were near 100 nM [20]. In agreement with these previous studies [20] we detected nanomolar levels of curcumin in the brain. We found that curcumin *in vivo *decreased several forms of mutant huntingtin aggregates visible with light microscopy in brain tissue. Curcumin can also inhibit the formation of fibrils of Abeta40 *in vitro *[26] and furthermore, it has been shown to cross cell membranes, and enter nuclei [60,61]. Thus, it is possible that it directly inhibited or slowed aggregate formation, resulting in the reduced density observed after *in vivo *curcumin treatment. However, our attempt to demonstrate a direct effect of curcumin on aggregate sizes in PC12 cells inducibly expressing exon 1 of mutant htt only revealed a minimal effect at low concentrations, while confirming an increase in aggregate size at μM concentrations. Although the rapid formation of aggregates *in vitro *clearly differs from the slow process occurring *in vivo *in brain, our data do not demonstrate conclusively that the effect of curcumin on aggregates is direct.

Several *in vitro *studies have shown that one form of aggregate, large inclusion bodies, are protective [62,63]. However, recent data suggest that reducing improper folding of monomeric htt may reduce both inclusion bodies and cell death [64]. Additionally, the speed of aggregate formation may predict their toxicity [65,66]. Thus, it is possible that reducing or preventing even the large "protective" aggregates, could be beneficial to the cell. To determine whether the effects of curcumin on aggregates have beneficial consequences for cellular functions, we also measured several striatal transcripts that are known to be affected by mutant huntingtin and which are markedly decreased in the striatum of control CAG 140 KI mice. In favor of a beneficial effect of curcumin on huntingtin pathology, several deficits in striatal transcripts in curcumin-fed KI mice were attenuated in this study. CB1 and D1 levels in curcumin-treated KIs were no longer different to control WTs, and furthermore, the transcripts encoding DARPP-32 and D1 receptors were significantly improved from control-fed KIs. Previously, we have demonstrated, using optical density, that DARPP-32 levels are normal at 4 m of age in these mice [11]. Therefore, we did not measure the effects of curcumin on this protein. It is not unusual for mRNA levels to decrease prior to protein levels [67] and indeed, by 12 m, we have found a profound loss of DARPP-32 protein (by optical density) in these mice [11]. In general, it appears that the mRNA levels are a more sensitive measure of neuronal dysfunction than protein levels at early stages of the disease in this slowly progressive mouse model.

In addition to protective effects of curcumin on pathology and transcription, lifelong treatment rescued deficits in rearing in the open field observed in these mice at 1 month of age and differences in climbing and the pole test normally observed between KI and WT mice were abolished by curcumin. The effects of treatment on behavioral deficits were more modest at 4.5 months, suggesting that most of the beneficial effect of curcumin may occur prior to or during the period of aggregate formation [12]. Some of the behavioral improvements observed at 4.5 months may be related to improvements in the level of the striatal transcripts for the D1 dopaminergic receptor and DARRP-32 as these molecules play a key functional role in subsets of striatal efferent neurons [68]. However, the modest improvement in other transcripts may explain the limited behavioral benefits observed. For example, decreases in CB1 mRNA in HD have recently been shown to be particularly critical for functional outcome [69].

Curcumin treatment had detrimental effects on some motor behaviors both in WT and KI mice. Specifically, climbing was affected by curcumin in WTs and rotarod performance in both genotypes. This effect was not due to the administration of curcumin during pre or early post-natal development because even when administered in adulthood for a similar length in time, curcumin impaired rotarod performance in 8 month old WT C57Bl/6 J mice of both sexes, and also impaired climbing in males. The detrimental effect of curcumin on motor behavior was not due to an effect on body weight since curcumin-treated mice had similar body weight to control-fed mice. Similarly, it is unlikely that this effect is related to a general muscle weakness because curcumin-treated adult WT mice initially showed increased grip strength, which then normalized. Activity and food utilization were unaffected by curcumin treatment, and we found no evidence of behavioral despair in the forced swim test. Intriguingly, previous studies have shown that the anti-oxidant CoQ impairs rotarod performance in WT mice [57], which we have also noted in WT littermates of CAG140 mice [56]. Other antioxidants like EGCG have been shown to interfere with climbing behavior by impacting the dopaminergic transmission [70]. Detrimental effects of anti-oxidants on mouse motor behavior could be related to their effects on redox balance which is important for several aspects of physiology including learning and memory, and normal cellular function and in particular autophagy, a clearance mechanism that may play a key role in HD pathophysiology [71]. This latter mechanism, however, is difficult to reconcile with our observation that curcumin treatment reduces htt aggregates in striatum. The significance of these adverse behavioral effects of both CoQ and curcumin for the potential therapeutic use of these compounds remains unclear. Indeed, CoQ has been used for many years in patients with Parkinson's disease and in clinical trials of HD for many years without adverse effects [72,73]. Furthermore, blood chemistries and the absence of significant adverse effects showed that curcumin was safe and well-tolerated in recent trials in AD and in the elderly [30,31,35]. Therefore, the detrimental effects observed in mice with these compounds are unlikely to be of clinical significance. Interestingly, curcumin tended to reduce transcripts in WT mice, but this did not reach significance. Most of the deleterious effects were in WT mice and they tended to be in the opposite direction to the effect on KIs, which may indicate that the agent affects WT physiology differently to the mutant physiology.

## Conclusions

In summary, in this first report of curcumin as a therapeutic *in vivo *for HD, we have found that curcumin ameliorated three aspects of HD in CAG140 KI mice, with the most notable effect on the htt aggregates. We also observed partial improvement of transcriptional deficits, and partial behavioral improvement. Despite the presence of some detrimental effects of curcumin treatment in both KI and WT mice of unknown significance for humans, further investigation of this compound for its use in HD is warranted.

## Methods and materials

### *In vitro *aggregate experiments

PC12 cells inducibly expressing EGFP-tagged exon 1 of mutant htt were a kind gift from Dr Erik Schweitzer (UCLA [42]). Cells were cultured in DMEM containing 5% horse serum, 5% calf serum and 1% L-glutamine (Fisher Scientific, Pittsburgh, PA), 1% penicillin/streptomycin and 1% geneticin (Invitrogen, Grand Island, NY) in a humidified atmosphere containing 9.5% CO_2 _at 37°C. Cells were cultured in collagen-coated T75s and plated for experiments onto poly-D-lysine-coated 96-well plates (20,000 per well, no cells were plated in the outermost wells; plates and flasks from BDBiosciences, San Jose CA). On the following day after plating, the cells were induced with 0.1 μM Tebufenozide, a kind gift from Dr Erik Schweitzer (UCLA), or treated with ethanol (vehicle) and also treated with curcumin (Sigma Aldrich, St. Louis, MO; 5 nM, 50 nM, 500 nM, 5 μM, 10 μM or 20 μM) or DMSO (Fisher Scientific; vehicle for curcumin) using dilutions of 1:1000 to prevent toxicity from the DMSO or ethanol. Four wells were used for each treatment (8 for control-treated induced cells), the positions of which were pseudorandomized between independent experiments (n = 4). Cells were cultured for 48 or 72 h and then the medium was removed, the cells were washed in warmed PBS and then fixed in cold 4% PFA for 30 mins. The cells were washed again and covered with fluorescent mounting medium and stored in the dark for analysis. For analysis of aggregates, photomicrographs, at 10× magnification, were taken and were analyzed using ImageJ. One photo, centered over the well, was taken per well. The mean size of all aggregates per field of view (FOV, i.e., 1 FOV per well) was calculated by ImageJ. This mean size was then expressed as a proportion of the mean aggregate size of all control-treated induced wells. These proportions were then used for the final quantification and statistical comparisons, such that each treatment group contained n = 4 replicates, each from an independent experiment.

### Mice, husbandry, treatment

All experiments were performed in accordance with the US Public Health Service *Guide for Care and Use of Laboratory Animals *and were approved by the Institutional Animal Care and Use Committee at UCLA. CAG140 mice were bred in house from heterozygous (Het) KI × Het pairings (non-sibling pairs only). Resultant WT and homozygous KI mice were used for behavioral analyses. Het progeny were used to monitor body weight only. Mice were N3 (B6) on a 129 Sv × C57BL/6 J background.

Breeding mice (5 males, 10 females per group) were fed normal chow (NIH-31 number 7013, Harlan Teklad, Indianapolis, IN) or the same chow containing 555 ppm curcumin (Sigma Aldrich, St. Louis, MO) for at least 1 week prior to breeding and throughout breeding. This regimen was chosen based on previously published preclinical trials in mouse models of Alzheimer's disease showing drug effects in brain, and measurable curcumin in brain parenchyma following oral administration [20,25,26]. Breeding pairs were checked daily for litters, and the number of pups in each litter was noted at birth and at weaning. In some cases, the number of pups increased from the number at birth and this is due to incomplete parturition at the time that the litter was first counted. Two rounds of breeding were used to generate mice for the preclinical trial, and equal numbers of litters were generated in both treatment groups (n = 18 per treatment group). Groups were matched for gender, and group sizes were n = 30 - 32 for WTs, and n = 16-17 for KIs. HET mice (n = 15-19) were used only to monitor body weight and curcumin brain levels, and were not used for any behavioral testing. Progeny were genotyped and weaned by 21 days and curcumin or control chow was continuously fed to the mice until 4.5 months of age. Body weight was recorded weekly. Tail samples were taken at the end of the experiment in order to confirm genotyping and to measure CAG repeat length. The mean CAG repeat length alleles, measured by Laragen Inc., Culver City, CA, using an ABI 3730 sequencer and Genemapper software, were 117 ± 1 (n = 50) in control diet-treated, and 113 ± 1.4 (n = 46) (mean ± sem) in curcumin-treated KI mice (frequency distributions not significantly different, Chi square independence: ns).

To control for effects of developmental exposure to curcumin, an additional trial was conducted in 39 adult WT C57Bl/6 J (B6) mice purchased from Jackson laboratories (Bar Harbor, ME). Following 1 week habituation to housing, the mice were divided into weight- and gender-matched control-fed groups (N = 10 males, N = 10 females) and curcumin-fed groups (N = 10 males, N = 9 females). Mice were fed normal or curcumin chow, as above, from 2.7 months of age until 8 months. Prior to start of trial, mice were tested for climbing, grip strength and body weight, and groups were balanced using these data.

Food and water were available ad lib, and stocks of chow were stored at 4°C. Mice were housed in a temperature and humidity controlled room, on a reverse light-dark cycle (11 am lights off, 11 pm lights on). For food utilization for the trial in adult mice, chow was weighed twice weekly and the amount used per mouse per day calculated.

### Tissue sample preparation for HPLC

Measurements of curcumin in brain tissue were carried out as described previously [20]. Briefly, fresh frozen (WT, n = 2 control, n = 3 curcumin; mice were from the behavior trial) or PBS-perfused and subsequently fresh frozen (HET, n = 4 control, n = 5 curcumin; HETs were treated in parallel to mice in the behavioral trial) hemispheres of brain from mice treated with control- or curcumin-containing food were weighed, then finely powdered and homogenized in 10 vol of 1 M ammonium acetate (pH 4.6). Mice were euthanized at the end of the trial thus following approximately 5.5 m of treatment, which includes gestation, weaning and up to approximately 4.5 m of age. Homogenates were extracted in 95% ethyl acetate/5% methanol and dried under a continuous flow of N_2_. Dried extracts were redissolved in acetonitrile/methanol/water/acetic acid (41/23/36/1, all by volume), and injected onto a reverse phase HPLC column (Supelco Ascentis Express C18, 150 × 2.1 mm) equilibrated in 10 mM ammonium acetate and eluted (100 μl/min) with an increasing concentration of acetonitrile/isopropanol. Samples were detected at 262 nm, using tetramethoxycurcumin as an internal standard. The effluent from the column was passed directly to an Ionspray™ ion source connected to a triple quadrupole mass spectrometer (PerkinElmer Life Sciences Sciex API III+). The retention times of curcumin and internal standard were 28.24 and 30.27 min, respectively.

### Neuropathological analysis

A subset of curcumin- and control-fed CAG140 KI mice (n = 6 per group) were anesthetized and perfused with 4% paraformaldehyde and 0.5% glutaraldehyde, their brains removed, post-fixed for 6-8 h in 4% paraformaldehyde, cryoprotected in 30% sucrose and frozen for use. Sagittal cryosections at the level of 1.32 mm and 2.28 mm lateral of the midline [47] were used for analysis. Tissue cryosections (35 μm) were stained with polyclonal EM48 (EM48; X-J Li, Emory University) as described in [12]. Briefly, sections were washed in 0.01 M PBS and then endogenous peroxidases were inactivated by incubating in 1% H_2_O_2 _and 0.5% Triton X-100 in PBS, for 20 min. Non-specific binding sites were then blocked by incubating sections for 30 min at room temperature in PBS containing 3% bovine serum albumin (BSA) and 2% normal goat serum (NGS). The primary antibody, EM48, was diluted (1:300) in PBS containing 3% BSA, 2% NGS, 0.08% sodium azide, and 0.2% Triton X-100 and sections were incubated overnight at room temperature. The following day the sections were washed in PBS and then incubated in biotinylated goat anti-rabbit antibody (1:200; Vector ABC Elite; Vector, Burlingame, CA) for 2 h at room temperature, washed and then reacted with avidin-biotin complex (Vector ABC Elite) in PBS containing 0.2% Triton X-100 for 2 h. Immunoreactivity was visualized by incubation in 0.03% 3-3-diaminobenzidine tetrahydrochloride (Sigma, St. Louis, MO) and 0.0006% H_2_O_2 _in 0.05 M Tris buffer, pH 7.6. After rinses in Tris buffer, the sections were dehydrated, defatted, and mounted with Eukitt (Calibrated Instruments, Hawthorne, NY). Control sections, processed in parallel, were incubated in the absence of the primary or secondary antibodies. No staining was noted in control sections (data not shown). Huntingtin-stained nuclei and aggregates were analyzed with Stereo Investigator 5.00 software (Microbrightfield, Colchester, VT). Briefly, the contours of the striatum were drawn at 5× magnification. The software then laid down a sampling grid of 200 × 200 μm, on which counting frames of 20 × 20 μm were placed. Counting frames were located on the top left corner of each sampling grid, thus allowing for unbiased sampling, and these counting frames were used for quantification of each type of aggregate per section. Quantification was done at 100× magnification, using a 1.4 NA lens and 1.4 NA oil condenser, with a DVC real-time digital camera. The mean number of 1) stained nuclei, 2) nuclei containing microaggregates, 3) nuclei containing inclusions and 4) neuropil aggregates, per counting frame, was calculated per striatal section, per mouse. These data were then used to generate group means. Microaggregates [12,74] were defined as numerous, small, nuclear puncta, similar to those previously observed in other models of HD [44-46].

### Quantitative real time PCR analysis

A subset of curcumin- and vehicle-treated CAG140 WT and KI mice (control-treated: WT n = 8, KI n = 7; curcumin-treated: WT n = 8, KI n = 7) were quickly decapitated and their brains frozen in powdered dry ice. Total RNA was purified from one striata of fresh frozen tissue using QiaGen RNeasy mini kit (protocol as described by manufacturer, QIAGEN Sciences, Maryland, USA). During the RNA extraction procedure DNAse 1 treatment was performed to remove contaminating genomic DNA (RNase-Free DNase Set, QIAGEN, Hilden, Germany). The Invitrogen ThermoScript RT-PCR System (Invitrogen, Carlsbad, CA, USA) was used for cDNA synthesis with oligo dT primers. The cDNA was then analyzed by quantitative real time PCR using a Roche LightCycler 480 (UCLA Genotyping and Sequencing Core). PCRs were performed using LightCycler FastStart DNA Master plus SYBR Green 1 kit (Roche Diagnostics, Mannheim, Germany). Each assay included: 1) a standard curve of five serial dilution points of control cDNA (mouse EST clone of appropriate fragment of the gene of interest (Invitrogen, CA), 2) sample cDNA, 3) no template control. All samples were run in triplicates. The PCR cycling parameters were: 95°C for 5 min (1 cyc); 95°C for 10 sec, 65°C for 10 sec, 72°C for 10 sec (40 cyc). A dissociation protocol was established at the end of each run to verify the presence of a single product. The relative expression of genes of interest was calculated from Ct values using the Pfaffl method [54]. PCR efficiencies of each primer pair were calculated from standard curve analysis and incorporated into relative quantification calculations. The endogenous control was HPRT, hypoxanthine phosphoribosyltransferase, which was reported to be unchanged in mouse and human microarray studies [50,75] and has been used in other previous RT-PCR studies examining transcript changes in HD [50-52]. Designed primers yielded a product of about 200 bp for each gene (Table 5).

**Table 5:** Sequences of primers used for mRNA quantification analysis

Gene	Primer sequence
HPRT	F: GAAGAGCTACTGTAATGATCAGTCAACGG
	
	R: GAGAGGTCCTTTTCACCAGCAAGC

Substance P	F: ACCCAAGCCTCAGCAGTTCTTTG
	
	R: TTCTGCATCGCGCTTCTTTCATA

DARPP-32	F: AAGGACCGCAAGAAGATTCAGTTCT
	
	R: CTCTCCAGAGGTTCTCTGATGTGGA

Dopamine receptor 1	F: AGAAGCAAATCCGGCGCATC
	
	R: GGAGCCAGCAGCACACGAATAC

Dopamine receptor 2	F: GGTCTACTCCTCCATCGTCTCGTTC
	
	R: TAACGGTGCAGAGTTTCATGTCCTC

Cannabinoid receptor 1	F: CGGCCTTGCAGATACCACCTTC
	
	R: GGAACCAACGGGGAGTTGTCTC

Preproenkephalin	F: CCTGAGATAGAGAAAAGATACGGG
	
	R: GATGTTTCGTCAGGAGAGATGAGG

### General health monitoring and behavioral testing

All analysis was carried out blinded to genotype and treatment. Mice were habituated to the testing rooms for 15-20 mins prior to all testing. Body weight was monitored in both trials and the amount of chow utilized was also quantified in the adult trial. Open field, pole task, and rotarod testing all took place in the dark phase, while climbing activity was recorded during the light phase [11,12]. For the life-time curcumin trial, CAG140 KI and WT mice were tested at 1 m of age in the open field, 1.5 months in the climbing test, and at 4 months in the pole test and the rotarod [11,12]. This schedule was chosen to avoid excessive repeated testing that could influence the progression of the disease. For the trial in adult WT mice, mice were tested 4.5 and 8 months of age in the climbing test and the rotarod, and at 8 months of age in the open field (for 15 mins daily over a period of 4 d). In addition, WT mice in the adult trial were also tested for grip strength, a measurement of forelimb muscle strength as described previously [76] at 3.5, 4.5 and 8 months of age. Finally, in this trial, WT mice were tested using the Porsolt swim task at 8 months [77]. They were also tested using the tail suspension task [78] however, several mice "escaped" the tail suspension task by climbing up their tail ([79] n = 2 male control, n = 5 female control, n = 1 male curcumin, n = 3 female curcumin), thus forced swim test data only were used.

For rearing activity mice were placed individually in the center of the open field (Truscan, Coulbourn Instruments, Allentown, PA) and video recorded for later analysis. Mice were tested in 2nd through 4th hours of the dark cycle, using a red light (25 W) for illumination. For climbing, mice were placed in a wire cylinder (3 ¾″ height × 4″ diameter) for 5 mins and their behavior recorded for later video analysis, as described previously [11]. For the pole task mice were placed head facing upwards on a vertical pole and trained to turn around and descend to the bottom of the pole, as described previously [11]. For rotarod performance mice were trained to walk on an accelerating rotating axle (Ugo Basile, Varese, Italy), as previously described with slight modifications [11]. Briefly, mice were given 3 trials/day over 4 days (5-40 rpm over 10 mins, with approximately 30 mins between successive trials) and the latency to fall was measured. For training the axle of the rotarod was covered with smooth rubber (smooth axle). On the 5th day mice received 1 trial at each of 10, 20 and 30 rpm (smooth axle) and on the final day mice received 1 trial at each of 20, 24, 30 and 36 rpm using a grooved axle. Testing was carried out approximately half way through the dark phase, under a red light (25 W). Any mice that clung to the axle for 3 successive rotations were removed and the time of removal recorded and used as the latency. The proportion of mice that cling is very small, thus we did not analyze these animals separately [11]. For the Porsolt swim task (adult trial only), mice were placed in 12 cm of water (temperature: 26°C) in a large Plexiglas beaker for 6 mins and their behavior was videotaped for analysis. Latency to become immobile and duration of immobility were quantified.

### Statistical analysis

GBstat (V8.0) and SAS (V 9.1) were used for statistical analyses. Comparisons of *in vitro *aggregate size proportions were completed using Kruskal-Wallis ANOVAs followed by Mann Whitney U tests. For *in vivo *data, outliers were detected using Grubbs test and removed from analyses. Measures of huntingtin pathology (immunostained nuclei and aggregates) were analyzed with completely randomized ANOVAs followed by Fisher's LSD for post hoc analysis. For qRT-PCR results, data were analyzed with completely randomized ANOVAs followed by Bonferroni t tests, corrected for 4 comparisons (control-fed WT V control-fed KI; control-fed WT V curcumin-fed WT; control-fed WT V curcumin-fed KI; control-fed KI V curcumin-fed KI). Correlations of mRNA data were carried out in GraphPad prism V4. Comparisons of husbandry data were made with Student's t tests or repeated measures ANOVA followed by Fisher's LSD post hoc tests. Body weights were analyzed using mixed generalized linear model ANOVAs in SAS using Bonferroni's adjusted Student's t-tests for post hoc analysis. Behavioral data and food intake were analyzed using one, two or three factor ANOVAs followed by Fisher's LSD for post hoc analysis. Significance was defined as *α *= 0.05 for all analyses.

## Abbreviations

HD: Huntington's disease; WT: Wildtype; KI: Homozygous knock in; Het: Heterozygous knock in; ns: Non-significant

## Competing interests

The authors declare that they have no competing interests.

## Authors' contributions

MAH participated in design and coordination of the study, performed *in vitro *experiments, behavioural studies, data analyses, and drafted the manuscript. CZ performed and analyzed the pathological studies. VM performed transcript analysis. RPL and SP participated in behavioural studies. NF participated in transcript analysis. PM and SAF carried out HPLC analyses and SAF participated in the design of the study and interpretation of the data and manuscript preparation. SZ assisted in interpretation and reviewed the manuscript. MSL participated in design and coordination of study and reviewed the manuscript. MFC conceived of study, participated in design and coordination, data interpretation, and in writing the manuscript. All authors read and approved the final manuscript.
